# Functional Capacity and Difficulties in Activities of Daily Living From a Cross-National Perspective

**DOI:** 10.1177/08982643221128929

**Published:** 2022-10-15

**Authors:** Tiia Kekäläinen, Martina Luchetti, Angelina Sutin, Antonio Terracciano

**Affiliations:** 1 Gerontology Research Center, Faculty of Sport and Health Sciences, 541605 University of Jyväskylä, Finland; 2 Department of Behavioral Sciences and Social Medicine, College of Medicine, 12236 Florida State University, Tallahassee, FL, USA; 3 Department of Geriatrics, College of Medicine, 12236 Florida State University, Tallahassee, FL, USA

**Keywords:** aging, disability, health, cognitive function, physical function

## Abstract

**Objectives**: This study investigated whether physical and cognitive functioning predicts developing difficulties in basic or instrumental activities of daily living (ADL/IADL), and whether country-level factors moderated the associations. **Methods**: 69,227 adults aged 50+ from 19 countries were followed for up to 14 years. Cox regression and meta-regression analyses were used. **Results**: Higher grip strength was associated with a 45% lower risk of developing ADL limitations and a 47% lower risk of IADL limitations. The corresponding values were 22% and 23% for peak flow, 20% and 23% for word recall, and 20% and 24% for temporal orientation. The associations were similar and statistically significant in most countries, but some associations were weaker in countries with lower GDP and lower service coverage. **Discussion**: Good physical and cognitive functional capacity protects from ADL and IADL limitations consistently across Western countries. The associations may be stronger in countries with more resources.

## Introduction

Activities of daily living (ADL) are activities needed to live independently in the community. These activities are classified into basic ADL and instrumental activities of daily living (IADL) (Katz, 1983; Lawton & Brody, 1969). ADLs are basic self-care activities required to take care of one’s physical needs, such as eating, dressing, and personal hygiene. IADLs are more complex activities that require planning and thinking, such as cooking, cleaning, shopping, and managing finances. Difficulties in ADLs and IADLs can be seen as an indicator of disability (Guralnik & Ferrucci, 2003), and they predict decreases in quality of life, comorbidity, and mortality (Millán-Calenti et al., 2010; Qiao et al., 2021).

The prevalence of difficulties in ADL and IADL increases with age and are typically first seen in more complex activities captured by IADL (Chatterji et al., 2015; Millán-Calenti et al., 2010). Both ADL and IADL require physical and cognitive functioning, and lower levels of physical and cognitive performance are risk factors for developing difficulties in ADL and IADL (Dodge et al., 2005; Duchowny et al., 2018; Makizako et al., 2015; McGrath et al., 2020; Vermeulen et al., 2011; Yam & Marsiske, 2013). Physical weakness has somewhat stronger associations with difficulties in ADL, whereas cognitive impairment is a risk factor especially for difficulties in IADL (McGrath et al., 2020). For cognitive functioning indicators, there is some evidence that poor baseline memory (Makizako et al., 2015; Yam & Marsiske, 2013) and poor performance in executive functions (Johnson et al., 2007) are more important predictors of ADL and IADL limitations than indicators of global cognitive functioning. In contrast, studies on physical functioning tend to find similar results regardless of indicator; for example, muscle strength, gait speed, peak flow, and balance (Vaz Fragoso et al., 2008; Vermeulen et al., 2011). However, previous studies were mainly based on relatively small single-country samples, and less is known from international, large-scale, prospective studies on physical and cognitive functions as risk factors for ADL and IADL.

Furthermore, disability is not solely a result of the individual’s characteristics, as the environment has a major effect especially on the experience of disability (Nagi, 1964; Verbrugge & Jette, 1994; World Health Organization, 2001). Difficulties in ADL and IADL occur when there is a gap between a person’s capability and the environment’s demands (Verbrugge & Jette, 1994). The effect of the environment is seen in wide differences in the prevalence of disability and functional limitations between countries. For example, older adults report more difficulties in the US than in Europe (Fernandes et al., 2008) and more in southern and eastern Europe than in northern and continental Europe (Jerez-Roig et al., 2018; Portela et al., 2020). Even though similar patterns of country differences are found for indicators of physical and cognitive performance (Andersen-Ranberg et al., 2009; Barbosa et al., 2021; Formanek et al., 2019), the differences between countries in ADL and IADL are not completely explained by differences in physical performance (Van Den Brink et al., 2003).

Disability is an outcome of the interaction between individual functional capacity and the environment (Guralnik & Ferrucci, 2003). The environment may weaken or strengthen the association between functional capacity and disability. Previous cross-sectional findings support the idea that the correlates of disability may differ between countries. A study among European countries showed that low cognitive performance, for example, was more common among older adults having difficulties in ADL in southern Europe than in other regions (Jerez-Roig et al., 2018). A study examining a small sample of Finnish, Dutch, and Italian men showed that the associations between physical performance and difficulties in ADL and IADL were similar between the three European countries but the associations were weaker compared to previous studies among US older adults (Van Den Brink et al., 2003). However, larger, systematic studies comparing country-level differences in the predictors of ADL and IADL are missing. This knowledge may help to understand who has the highest risk for developing disability in different countries.

In addition, the moderator role of country-level environmental factors on the association between functional capacity and disability is not clear. Both economic situation and service access may moderate the role of individual characteristics. A study focusing on within-country regional differences showed that individual characteristics have a larger role in end-of-life well-being in areas with higher unemployment and poor service access (Gerstorf et al., 2010). A country’s economic situation and health policies are likely to be reflected in various socioeconomic indicators (Cameron et al., 2013) that may shape the association between functional capacity and limitations in ADL and IADL. Thus, in the present study, we focus on the major country-level economic and health indicators, specifically gross domestic product (GDP) per capita, health expenditure as a share of GDP, and the Universal Health Coverage (UHC) service coverage index (SCI) (World Health Organization, 2019). These indicators capture a country’s overall economic situation, the standard of living, and public health policies (Sposato & Saposnik, 2012) as well as coverage of essential health services (World Health Organization, 2019) that are likely to help maintain independence in ADL and IADL. Focusing on cross-level interactions may help to understand the interplay between individual and environmental characteristics in the disability process.

The purpose of this study was to investigate whether physical and cognitive performance predict difficulties in ADL and IADL similarly across Western countries. This work contributes to existing knowledge of the association between functional capacity and ADL/IADL limitations by providing a large longitudinal cross-national comparison based on harmonized measures across 17 European countries, Israel, and the US This study further contributes to this research area by comparing the predictive value of four functional capacity indicators (grip strength, peak flow, word recall, and temporal orientation) on two outcomes (ADL and IADL limitations) and investigates country-level differences and country-level moderators of the association between functional capacity and ADL/IADL limitations.

Based on past research (e.g., McGrath et al., 2020; Vermeulen et al., 2011), we expected both physical and cognitive functioning to be associated with ADL and IADL limitations, with stronger associations between physical functioning and ADL and cognitive functioning and IADL. The cross-country comparison, however, was exploratory due to the lack of previous comparable studies. On the one hand, functional capacity may have a weaker role in the experience of disability in countries with a more supportive environment in terms of economics, social policy, and service access. Based on this hypothesis, the association between physical and cognitive performance and difficulties in ADL and IADL would be weaker in wealthier countries with better health care services. On the other hand, in less wealthy countries, the higher prevalence of difficulties may be partly due to a more demanding environment that even relatively good functional capacity does not protect from experiencing difficulties. Based on this alternative hypothesis, the association between physical and cognitive performance with difficulties in ADL and IADL would be weaker in countries with less economic resources and health care services.

## Methods

### Participants

Data were from two large aging studies, the Survey of Health, Ageing and Retirement in Europe (SHARE) (Börsch-Supan et al., 2013) and the Health and Retirement Study (HRS) (Sonnega et al., 2014). SHARE and HRS are sister-studies with data collection and measures harmonized to enable cross-national comparisons. In both surveys, the target sample were all community-dwelling persons over age 50 years, and their spouses. All participants interviewed in any wave were part of the longitudinal follow-up and were contacted to participate regardless of institutionalization status. In addition, end-of-life/exit interviews on deceased participants were conducted with relatives or other persons close to the deceased to gain information on the cause of death and circumstances at the end of their life, including difficulties in daily activities.

To maximize the number of included countries and harmonized measures between SHARE and HRS and have a long enough follow-up, waves 2 (collected in 2006, DOI: 10.6103/SHARE.w2.800) and 4 (2010, DOI: 10.6103/SHARE.w4.800) were used as a baseline for SHARE. 15 countries (Austria, Belgium, Switzerland, Germany, Denmark, Spain, Ireland, France, Greece, Italy, Israel, Czech Republic, Sweden, Poland, and Netherlands) participated in wave 2 and 4 more countries (Estonia, Hungary, Portugal, and Slovenia) were included in wave 4. Ireland did not participate in later SHARE waves and thus was excluded from the present sample. Follow-up information from all available waves was utilized: wave 4 (for those with baseline in wave 2), wave 5 (2013, DOI: 10.6103/SHARE.w5.800), 6 (2015, DOI: 10.6103/SHARE.w6.800), 7 (2017, DOI: 10.6103/SHARE.w7.800), and 8 (2019–2020, DOI: 10.6103/SHARE.w8.800). Release 8.0.0. was used (SHARE Release Guide 8.0.0., 2022). Data are available in a public, open access repository for registered users (http://www.share-project.org/home0.html). The SHARE study was reviewed and approved by the Ethics Committee of the University of Mannheim in waves 2 and 4 and from wave 4 onwards by the Ethics Council of the Max Planck Society. All participants provided written informed consent.

In HRS, waves 8 (2006) and 9 (2008) were used as the baseline. Physical measurements were conducted for a random one-half of the sample in 2006 and in 2008 for the other half of the sample. These two samples were combined as the baseline. These measurements were introduced in 2004 but were not used for baseline because they were only available for a small random subsample. Follow-up information from all available waves was utilized: wave 9 (for those with baseline in wave 8), wave 10 (2010), 11 (2012), 12 (2014), 13 (2016), 14 (2018), and 15 (2020). The public release data are available for registered users (https://hrs.isr.umich.edu/about). Ethical approval for the HRS was obtained from the University of Michigan Institutional Review Board. All participants provided either oral (interviews) or written (physical measures) informed consent.

**Figure 1.**
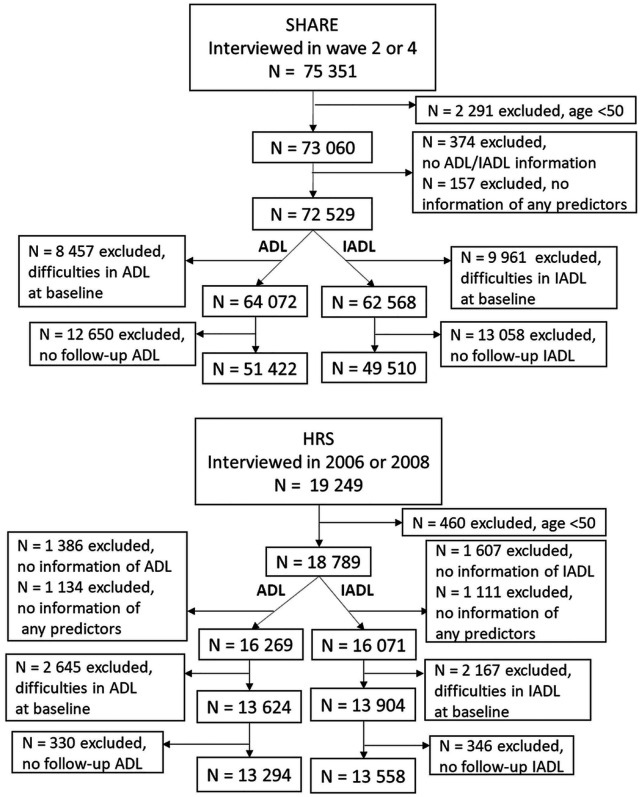
Flow of the Participants.

The flow of selected participants for each study is shown in Figure 1. Inclusion criteria were (1) 50 or older at baseline, (2) information about ADL/IADL and at least one physical and cognitive function predictor (i.e., grip strength, peak flow, temporal orientation, and word recall) available at the baseline, (3) no difficulties in ADL or IADL at baseline, and (4) at least one follow-up on ADL or IADL available either from the core interview or exit/end-of-life interview. The participants for ADL and IADL analyses were selected separately because the number of missing values and the prevalence of difficulties differs between the ADL and IADL scales.

In SHARE, a total of 75,351 participants were interviewed in either wave 2 or wave 4 from the 17 selected countries, and 73,060 were 50 or older at the time of the interview (Figure 1). This sample included 35,072 participants who participated for the first time in wave 2, 23,620 participants from wave 2 countries who participated for the first time in wave 4, and 14,368 participants from countries who participated for the first time in wave 4. The final sample size was 51,422 participants for ADL (no difficulties in ADLs at baseline and had follow-up assessments) and 49,510 participants for IADL (no difficulties in IADLs at baseline and had follow-up assessments), for a total of 54,260 participants. In HRS, a total of 19,249 men and women participated in an enhanced face-to-face interview including physical measures in either 2006 or 2008, and 18,789 of them were 50 or older. The final sample size was 13,294 participants for ADL (no difficulties ADLs at baseline and had follow-up assessments) and 13,558 for IADL (no difficulties IADLs at baseline and had follow-up assessments), for a total of 14,553 participants.

### Variables

#### ADL and IADL

Modified versions (Marmot et al., 2003) of the ADL index (Katz, 1983) and IADL scale (Lawton & Brody, 1969) were used to assess perceived difficulties in different activities. These questionnaires have good measurement equivalence between HRS and SHARE (Chan et al., 2012). Participants were asked to report if they had difficulties in different activities because of a physical, mental, emotional, or memory problem. Participants were told to exclude difficulties that were expected to last less than 3 months. ADL was assessed by six tasks (dressing, walking across a room, bathing or showering, eating, getting in or out of bed, and using a toilet) and IADL was assessed with six tasks (using a map, preparing a hot meal, shopping, making telephone calls, taking medications, and managing money). A binary variable that indicated difficulties with any ADL was computed (0 = No difficulties, 1 = Difficulties in at least one item). A separate binary variable was similarly computed for IADL (0 = No difficulties, 1 = Difficulties in at least one item). Difficulties in ADL and IADL in the last 12 months (SHARE) or 3 months (HRS) of life were asked in end-of-life/exit interviews from a relative or other person close to the deceased. This information obtained from the end-of-life/exit interviews was included in the follow-up analyses.

#### Cognitive functioning

Cognitive functioning was assessed by memory performance (a 10-word recall test) and temporal orientation. In the *10-word recall test* (Brandt et al., 1988; Harris & Dowson, 1982), participants were asked to listen carefully to a list of 10 words read out loud. Participants were asked to recall as many words as they could remember immediately (immediate recall) and after a short interval (delayed recall). The sum of correctly remembered words on immediate and delayed recall was calculated. *Temporal orientation* was assessed by questions about the current date, month, year, and day of the week. A binary variable was generated with categories 0 = Poor temporal orientation (Incorrect response to at least one question) and 1 = Normal temporal orientation (All four questions answered correctly).

#### Physical functioning

Physical functioning was assessed by *grip strength* and *peak flow*. *Grip strength* was measured using a handheld dynamometer (Smedley, S Dynamometer, TTM, Tokyo, 100 kg) twice from both hands, alternating between hands (Andersen-Ranberg et al., 2009; Crimmins et al., 2008). Participants were instructed to stand (if possible, otherwise seated) with their arm at their side at a 90-degree angle. The maximum result was used in the analyses. *Peak flow* was assessed with Mini-Wright Peak Flow Meter (Wright, 1978). Participants were instructed to blow as hard and as fast as possible into the mouthpiece of the device in a standing position after taking a deep breath (Crimmins et al., 2008; Mehrbrodt et al., 2019). Two measurements were taken in SHARE and three in HRS and the best result across the trials was used in the analyses. In both studies, values less than 60 L/minute were coded to 60 and values over the last tick of the device were coded to 890 L/minute.

#### Covariates

Included age in years, gender (0 = male, 1 = female), depression, chronic diseases, education, marital status, height, and weight. Depression was a binary variable (0 = No and 1 = Yes) that indicated elevated depressive symptoms on the cut-point for the EURO-D scale in SHARE (score 0–3 = Not depressed, score 4–12 = Yes) (Prince et al., 1999) and the 8-item version of the CES-D in HRS (score 0–2 = not depressed, score 3–8 = yes) (Radloff, 1977). The number of chronic diseases was calculated based on whether participants reported that a doctor had diagnosed them with heart disease, high blood pressure, stroke, diabetes, chronic lung disease, arthritis, cancer, hip fracture, or serious memory disease. The international standard classification of education (ISCED) with six categories was used in SHARE (UNESCO Institute for Statistics, 2006) and a classification with seven categories from “No degree” to “Professional degree” in HRS. Marital status was coded to Married or in a registered partnership = 1 and Other = 0 (includes never married/single, divorced, widowed, and other). Height and weight were self-reported.

#### Country-level indicators

Gross domestic product (GDP) per capita, health expenditure (% of GDP), the UHC service coverage index (SDG 3.8.1.) indicators on service capacity and access, and geographic area were used as country-level indicators. GDP and health expenditure from the baseline year 2006 and service capacity and access from the year 2010 (available only every 5 years) were collected from the World Bank. GDP per capita is a country’s GDP divided by its total population and is an indicator of national wealth. Health expenditure as a share of GDP is the ratio of spending on health care goods and services compared to total spending in the economy. UHC SDG index indicates the average coverage of four essential health service areas needed by most populations: reproductive, maternal, newborn, and child health, infectious diseases, non-communicable diseases, and service capacity and access (World Health Organization, 2019). The service capacity and access-area includes information about hospital access (hospital beds per capita), health workforce (health professionals per capita), and health security (International Health Regulations core capacity index) (World Health Organization, 2019). The grouping of countries to geographic areas was done in line with previous studies based on SHARE data (e.g., Scheel-Hincke et al., 2020): northern Europe (Denmark and Sweden), central Europe (Austria, Belgium, France, Germany, Netherlands, and Switzerland), southern Europe and Israel (Greece, Israel, Italy, Portugal, and Spain) and eastern Europe (Czech Republic, Estonia, Hungary, Poland, and Slovenia).

### Statistical Analyses

Attrition analysis was performed using independent samples t-test and x^2^-test to compare the participants with and without follow-up information. Binary logistic regression analyses were used to test whether physical and cognitive performance at baseline predicted the likelihood of available follow-up information after adjusting for gender.

Cox regression hazard models were used to test whether physical and cognitive performance at baseline predicted incidence of difficulties in ADL or IADL over up to 14 years of follow-up in each country. Identical analyses for difficulties in ADL and IADL as an outcome were conducted and the predictors (grip strength, peak flow, word recall, and temporal orientation) were tested in separate models. Time was coded in years from the baseline assessment as year-to-incidence in difficulties in ADL or IADL. For participants who did not develop difficulties during the follow-up, cases were censored at their last available assessment. For participants who died during the follow-up and had the end-of-life/exit interview available that indicated the absence of difficulties before death, cases were censored at their year of death. Z-scores for grip strength, peak flow, and word recall separately for each country were calculated and used in cox regression to facilitate interpretation of hazard ratios.

The proportional hazard assumption was checked with Kaplan–Meier plots and interaction terms between time and predictors in time-dependent covariate Cox regression models. As the incidence of both ADL and IADL exponentially increases with age, age violated the assumption. Thus, all Cox regression models were stratified by age group (50–64, 65–79, and 80+) to allow separate baseline hazard functions to be fitted within different age strata. The associations of the predictors were still modeled as a single set of common effects across strata. All Cox regression models were adjusted for baseline age, gender, education, marital status, depressive symptoms, and chronic diseases. The models with grip strength were also adjusted with weight and height, and models with peak flow with height.

Results of each country (hazard ratios with 95% confidence intervals) were combined using random-effects meta-analysis to estimate the overall effect. Forest plots were used to visualize results. Random-effects meta-regression was used to assess the country-level moderators (GDP, health expenditure, service capacity and access, and geographic area) and were tested in separate models to avoid multicollinearity. Dummy-coded variables for geographic area (northern Europe vs. others, central Europe vs. others, southern Europe vs. others, and eastern Europe vs. others) were used in the meta-regression. I^2^ was used as an indicator of heterogeneity with values below 30 indicating low and values above 50 indicating notably heterogeneity (Higgins & Thompson, 2002). Additional analyses were performed to test whether the drop-out rate moderated the strength of the associations.

IBM SPSS Statistics Version 26 was used for descriptive statistics and Cox regression and Stata Version 16 was used for the meta-analysis.

## Results

Descriptive statistics are in Table 1. Over the up to 14 years follow-up, on average, 28% of participants developed difficulties in ADL (range 12.0–44.5%) during their 64,716 person-years of follow-up and 29% of participants developed difficulties in IADL (range 14.2–44.5%) during their 63,067 person-years of follow-up.

**Table 1.** Descriptive Statistics for the Full Sample.

Country	N	Age		Educa-tion		Female	Married	Grip Strength		Peak Flow		Word Recall		Normal Orien-tation	ADL Diff	IADL Diff	GDP	Health Exp	Service Cap
		M	SD	M	SD	%	%	M	SD	M	SD	M	SD	%	%	%			
SHARE: Austria	4315	64.8	9.3	3.2	1.3	57.1	64.4	35.1	11.8	362.6	156.7	9.9	3.8	91.6	19.1	22.6	40.6	9.5	83.9
SHARE: Belgium	4455	63.1	9.8	3.0	1.5	53.5	70.0	36.3	12.1	367.2	157.3	9.4	3.6	85.5	26.0	23.8	38.7	9.2	92.8
SHARE: Czechia	4655	64.3	8.9	2.6	1.2	57.1	67.8	35.6	11.4	377.4	199.6	9.0	3.3	86.9	27.0	27.8	15.3	6.2	87.5
SHARE: Denmark	2402	62.2	9.9	3.5	1.4	52.2	71.2	36.6	12.8	417.2	148.9	10.4	3.3	87.7	23.1	22.3	52.0	9.2	98.7
SHARE: Estonia	5659	65.6	9.5	3.3	1.2	59.4	64.1	34.0	12.2	349.9	141.9	8.9	3.7	89.6	28.2	32.1	12.6	4.8	90.5
SHARE: France	4725	64.2	10.1	2.5	1.7	55.7	66.5	33.8	11.9	367.6	155.1	8.8	3.6	85.0	24.5	21.8	36.4	10.4	93.6
SHARE: Germany	1565	63.7	8.5	3.5	1.1	52.1	80.4	37.6	11.5	375.3	148.3	9.8	3.1	91.7	25.0	21.8	36.3	10.2	96.9
SHARE: Greece	2396	64.4	9.7	2.1	1.6	53.9	74.7	33.8	11.5	348.6	124.8	8.1	3.0	93.4	18.5	28.0	24.8	8.3	82.9
SHARE: Hungary	1718	64.0	8.6	3.1	1.0	56.9	69.7	33.4	12.3	289.4	135.5	9.0	3.4	83.9	18.9	24.1	11.5	7.8	87.5
SHARE: Israel	1866	66.5	8.8	3.0	1.6	56.0	80.8	30.5	10.6	309.1	133.9	8.1	3.4	84.3	23.9	28.3	21.9	6.9	96.9
SHARE: Italy	3281	64.1	8.8	2.0	1.2	52.8	83.6	33.8	11.4	318.4	171.5	7.9	3.4	88.7	27.0	24.8	33.5	8.4	94.7
SHARE: Netherlands	2642	63.2	9.1	2.9	1.4	54.2	81.3	36.6	11.5	397.2	149.4	10.0	3.3	86.7	12.0	14.2	44.9	9.1	96.2
SHARE: Poland	1732	62.4	9.0	2.4	1.3	53.5	79.5	34.9	11.6	317.9	141.4	7.5	3.3	88.2	29.5	29.2	9.0	5.8	87.1
SHARE: Portugal	1471	63.9	8.9	1.7	1.4	53.7	82.4	30.8	10.8	290.2	183.0	7.6	3.4	89.1	29.9	22.8	19.8	9.4	91.7
SHARE: Slovenia	2214	65.1	9.8	2.9	1.2	57.0	74.6	34.6	12.2	361.2	169.3	8.2	3.6	82.6	21.7	25.2	19.7	7.8	93.6
SHARE: Spain	3528	65.5	10.1	1.6	1.4	52.9	80.7	30.4	11.4	289.3	197.5	6.8	3.3	79.2	30.5	36.5	28.4	7.8	94.4
SHARE: Sweden	2399	66.1	9.3	2.8	1.6	53.2	77.3	36.7	12.1	420.1	140.0	9.8	3.3	90.6	26.6	22.6	46.6	8.1	97.3
SHARE: Switzerland	3237	64.0	9.8	3.2	1.1	53.3	72.2	35.6	11.4	395.2	150.6	10.2	3.4	92.5	16.6	15.6	59.3	9.5	94.4
HRS: USA	14 967	68.2	9.9	2.2	1.5	58.1	63.2	31.9	11.1	363.2	133.8	9.8	3.4	78.4	44.5	44.5	46.3	14.7	96.9
Total	69,227	65.2	9.5	2.6	1.4	55.7	70.7	34.0	11.6	358.1	152.9	9.1	3.4	85.6	28.2	29.1	31.5	8.6	92.5

Note: Unadjusted mean and standard deviations or percentages are reported. The full sample includes participants who were included in either ADL or IADL analyses or both. ADL/IADL diff. = difficulties during the follow-up, Health exp. = Health expenditure, Service cap. =Service capacity and access.

The results of attrition analysis are shown in Supplementary Tables S1-S3 separately for each country. The availability of follow-up information was on average 83.3% (range 60.1–97.6%) for ADL and 82.5% (range 60.6–97.5%) for IADL. In general, follow-up information was available from older and less educated participants, and women were more likely to have available follow-up information on ADL and IADL than men (Table S1). These differences were not consistent across countries. After adjusting for gender (Table S2 and S3), participants with better functional capacity tended to have available follow-up information on both ADL and IADL. These associations were not consistent across countries and functional capacity indicators.

**Figure 2.**
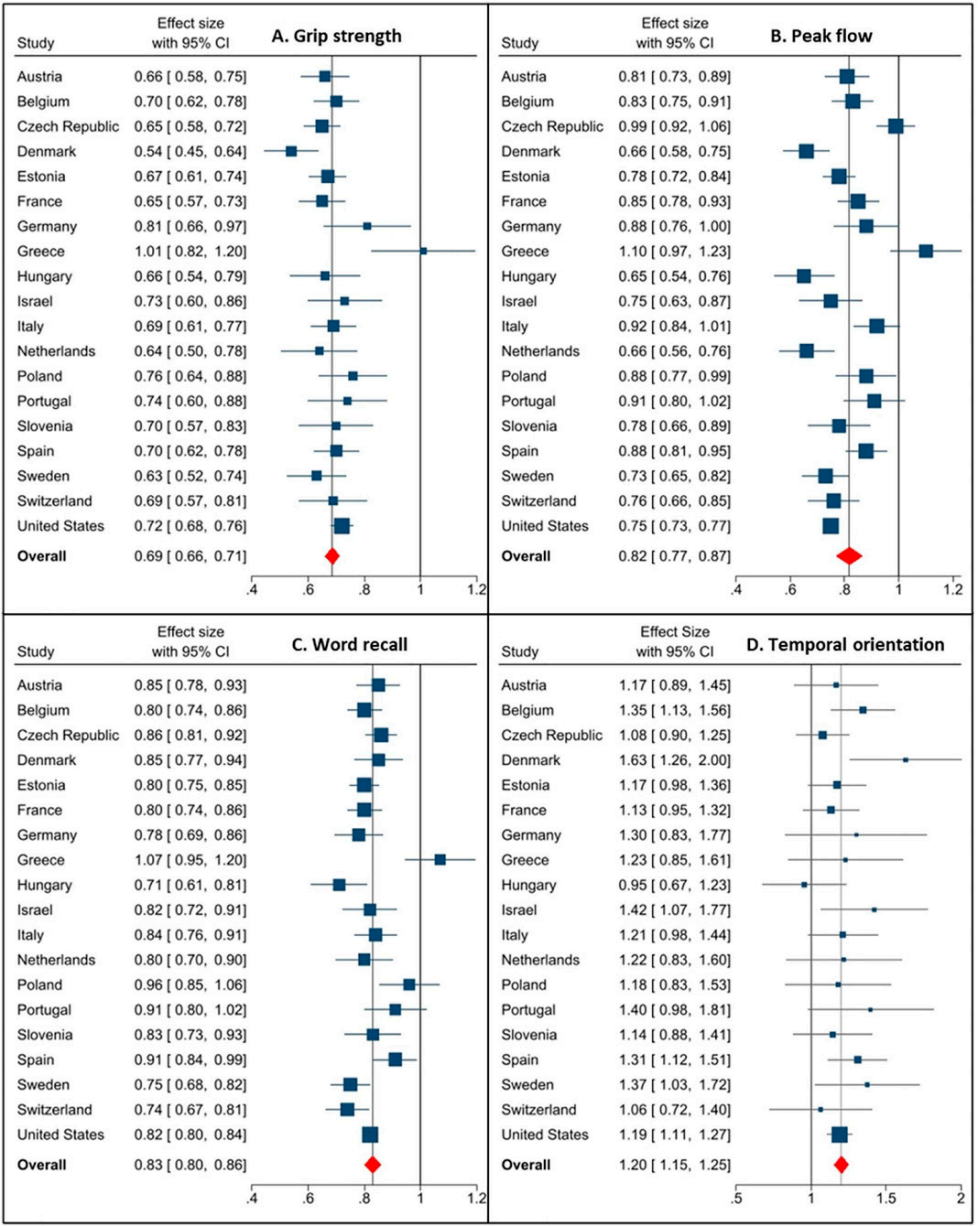
Association Between Grip Strength (a), Peak Flow (b), Word Recall (c), and Temporal Orientation (d) and ADL Limitations. Hazard Ratios With 95% Confidence Intervals Adjusted for Baseline Age, Gender, Education, Marital Status, Chronic Diseases, Depression, Height (Grip Strength and Peak Flow), And Weight (Grip Strength). The Marker Sizes are Proportional to the Study Weights.

**Figure 3.**
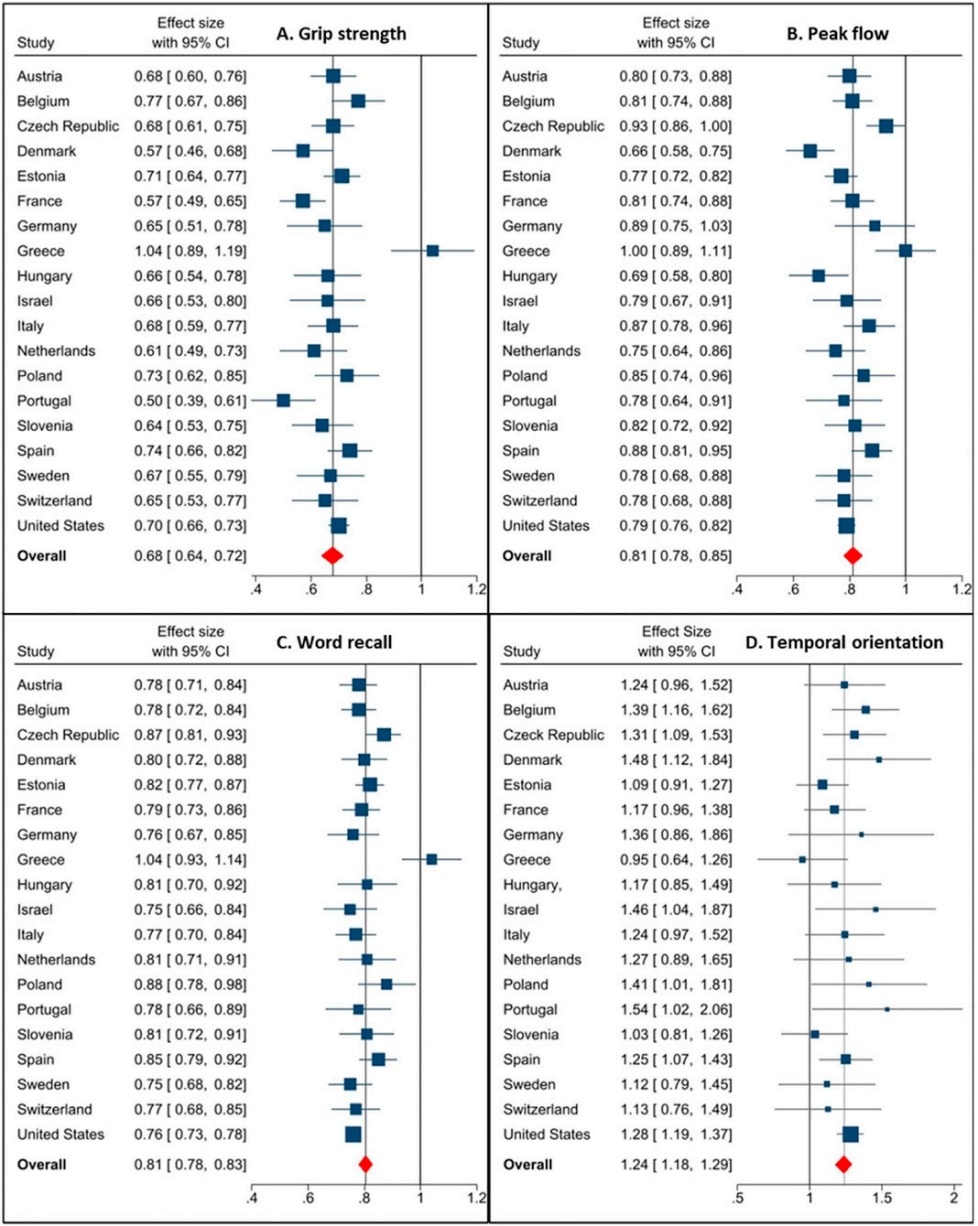
Association Between Grip Strength (a), Peak Flow (b), Word Recall (c), and Temporal Orientation (d) and IADL Limitations. Hazard Ratios With 95% Confidence Intervals Adjusted for Baseline Age, Gender, Education, Marital Status, Chronic Diseases, Depression, Height (Grip Strength and Peak Flow), and Weight (Grip Strength). The Marker Sizes are Proportional to the Study Weights.

The results of Cox regression analyses and meta-analyses are reported in Figure 2 and Supplementary table S4 for ADL and in Figure 3 and Supplementary table S5 for IADL. Weaker grip strength and peak flow, worse word recall performance, and poor temporal orientation were associated with developing difficulties in ADL and IADL over the follow-up in most countries and the meta-analysis. One positive SD from the mean grip strength was associated with a 45% lower risk of developing ADL difficulties and a 47% lower risk of developing IADL difficulties during the follow-up. The corresponding values were 22% and 23% for peak flow and 20% and 23% for word recall. Participants with poor temporal orientation at baseline had a 20% higher risk of developing difficulties in ADL and a 24% higher risk of developing difficulties in IADL during the follow-up.

Grip strength had a statistically significant association with ADL and IADL in 18/19 countries. The proportions were similar for peak flow (14/19 with ADL and 16/19 with IADL) and word recall (16/19 and 18/19, respectively). The association with temporal orientation was less consistent between countries and was statistically significant for only 8/19 countries for ADL and 6/19 countries for IADL. The pattern of associations was similar across countries (Figures 2 and 3) with one exception. None of the predictors had a statistically significant association with developing difficulties in ADL or IADL in Greece.

The I^2^ statistics suggested low heterogeneity for the association between temporal orientation and ADL (I^2^ = 0%) and IADL (I^2^ = 8.0%) and moderate heterogeneity for the association between grip strength and ADL (I^2^ = 32.1%). All other associations had notable heterogeneity: grip strength and IADL I^2^ = 73.1%, peak flow and ADL I^2^ = 86.7%, peak flow and IADL I^2^ = 71.5%, word recall and ADL I^2^ = 69.7%, and word recall and IADL I^2^ = 61.4%. As Greece was an outlier with nonsignificant associations between all predictors and ADL and IADL difficulties, sensitivity analyses without Greece were performed. The results of meta-analysis remained the same (Supplementary tables S4 and S5): associations of physical and cognitive indicators with ADL and IADL limitations were just slightly stronger and I^2^ suggested slightly lower heterogeneity between countries.

Moderation analyses showed some country-level moderator effects (Table 2). Service capacity and access had statistically significant moderator effects on the association between grip strength and IADL (Coefficient = −0.009, 95%CI = −0.018; −0.001), peak flow and ADL (Coefficient = −0.012, 95%CI = −0.022; −0.003), peak flow and IADL (Coefficient = −0.007, 95%CI = −0.014; −0.0001), word recall and ADL (Coefficient = −0.007, 95%CI = −0.013; −0.001), and word recall and IADL (Coefficient = −0.008, 95%CI = −0.012; −0.003), suggesting stronger associations in countries with higher service capacity and access. GDP had a statistically significant moderator effect on the association between word recall and IADL (Coefficient = −0.002, 95%CI = −0.003; −0.001), suggesting a stronger association in countries with higher GDP. Geographic area moderated the association between grip strength and temporal orientation and ADL. The predictive value of grip strength (Coefficient = −0.139, 95%CI = −0.220; −0.058) and temporal orientation (Coefficient = 0.306, 95%CI = 0.039; 0.571) was stronger in northern European countries (Denmark and Sweden) compared to other countries. The drop-out rate did not moderate the strength of the associations (*p* > .05, Supplementary Table S6).

**Table 2.** The Results of Country-Level Moderator Analyses.

	ADL		IADL	
	Coefficient	95%CI	Coefficient	95%CI
Grip strength				
GDP	−0.001	−0.003; 0.001	−0.001	−0.004; 0.002
Health expenditure	0.003	−0.007 0.014	−0.006	−0.025; 0.012
Service capacity and access	−0.004	−0.010; 0.003	−0.009*	−0.018; −0.001
Geographic area: Northern	−0.139**	−0.220; −0.058	−0.081	−0.309; 0.146
Geographic area: Central	−0.041	−0.097; 0.016	−0.045	−0.238; 0.149
Geographic area: Eastern	−0.046	−0.102; 0.010	−0.015	−0.211; 0.181
Geographic area: Southern	−0.005	−0.057; 0.067	0.014	−0.184; 0.211
Peak flow				
GDP	−0.003	−0.006; 0.000	−0.002	−0.004; 0.001
Health expenditure	−0.010	−0.003; 0.013	−0.005	−0.021; 0.010
Service capacity and access	−0.012*	−0.022; −0.003	−0.007*	−0.014; −0.000
Geographic area: Northern	−0.051	−0.272; 0.169	−0.067	−0.217; 0.083
Geographic area: Central	0.053	−0.138; 0.245	0.019	−0.108; 0.145
Geographic area: Eastern	0.077	−0.118; 0.272	0.029	−0.099; 0.157
Geographic area: Southern	0.123	−0.033; 0.359	0.086	−0.044; 0.216
Word recall				
GDP	−0.002	−0.004; 0.000	−0.002**	−0.003; −0.001
Health expenditure	−0.005	−0.019; 0.008	−0.009	−0.018; 0.000
Service capacity and access	−0.007*	−0.013; −0.001	−0.008**	−0.012; −0.003
Geographic area: Northern	−0.017	−0.147; 0.114	0.007	−0.101; 0.116
Geographic area: Central	−0.020	−0.129; 0.089	0.017	−0.071–0.105
Geographic area: Eastern	0.015	−0.096; 0.127	0.073	−0.018; 0.164
Geographic area: Southern	0.081	−0.033; 0.195	0.067	−0.026; 0.159
Temporal orientation				
GDP	0.002	−0.002; 0.006	0.003	−0.001; 0.007
Health expenditure	0.001	−0.016; 0.018	0.010	−0.004; 0.025
Service capacity and access	0.013	−0.001; 0.026	0.010	−0.003; 0.023
Geographic area: Northern	0.306*	0.039; 0.571	0.004	−0.277; 0.285
Geographic area: Central	0.014	−0.123; 0.150	−0.026	−0.202; 0.150
Geographic area: Eastern	−0.084	−0.215; 0.048	−0.116	−0.286; 0.054
Geographic area: Southern	0.105	0.044; 0.253	−0.047	−0.230; 0.137

Note: Coefficients are the interaction between the country-level factor and the predictor (grip strength, peak flow, word recall, or temporal orientation) on each outcome (ADL or IADL). CI = Confidence interval, *p < .05, **p < .01

Sensitivity analyses without Greece showed that most moderator effects of service capacity and access were not statistically significant after the exclusion of Greece (Supplementary Table S7). The moderator effect of service capacity and access on the associations between grip strength and IADL (Coefficient = −0.003, 95%CI = −0.008; 0.005), peak flow and ADL (Coefficient = −0.008, 95%CI = −0.018; 0.002), peak flow and IADL (Coefficient = −0.004, 95%CI = −0.011; 0.004), and word recall and ADL (Coefficient = −0.003, 95%CI = −0.009; −0.002) were not statistically significant after exclusion of Greece. The moderator effects of service capacity and access and GDP on the association between word recall and IADL (shown in Supplementary figure S1) as well as the moderator effect of northern Europe on the associations between grip strength and ADL and temporal orientation and ADL remained statistically significant. In addition, some new moderator effects were found when Greece was excluded. Health expenditure (Coefficient = −0.007, 95%CI = −0.011; −0.003) had a statistically significant moderator effect on the association between word recall and IADL and service capacity and access on the association between temporal orientation and ADL (Coefficient = 0.015, 95%CI = 0.0002; 0.030). These effects suggested stronger associations in countries with higher health expenditure and higher service capacity and access. In addition to the above-mentioned stronger associations in northern European countries that were found with the whole sample including Greece, geographic area moderated the association between word recall and IADL when Greece was excluded: the predictive value of word recall was weaker in eastern European countries compared to others (Coefficient = 0.073, 95%CI = 0.034; 0.112).

## Discussion

The aim of this study was to investigate whether physical and cognitive performance predicted incident difficulties in ADL and IADL, and whether the associations differed across countries with a large multi-national sample. Weaker grip strength and peak flow as well as worse word recall performance were associated with increased risk of developing difficulties in ADL and IADL in the meta-analysis and in most of the countries examined in the study. Difficulties in temporal orientation were associated with increased risk for ADL and IADL difficulties in the meta-analysis but the association was found in less than half the individual countries. In general, the results were consistent across Western countries. The few country-level moderators indicate a stronger association between functional capacity and developing difficulties in ADL or IADL in countries with better resources.

These findings broadly support previous work in this area that linked worse functional capacity to increased risk of developing difficulties in ADL or IADL (Dodge et al., 2005; Duchowny et al., 2018; Makizako et al., 2015; McGrath et al., 2020; Yam & Marsiske, 2013). In contrast to earlier findings (McGrath et al., 2020), no evidence of stronger associations between physical functioning and ADL difficulties and cognitive functioning and IADL difficulties was found. Weak muscle strength, poor pulmonary function, and poor memory are risk factors for limitations in both basic and instrumental daily activities. Of these functional capacity indicators, temporal orientation had the least consistent association. This may be because incorrect answers to temporal orientations questions were relatively rare (14% on average) in the sample free of difficulties in ADL or IADL at baseline. Grip strength seems to have the strongest predictive value for developing difficulties followed by a relatively similar size of the predictive value of peak flow and word recall. Recent studies have argued that while cognitive performance has improved among adults and older adults (e.g., Beller et al., 2022) and the same is the case for grip strength among the oldest population (80+), average grip strength has stagnated or even decreased among middle-aged adults (Beller et al., 2019). This is in line with the finding that middle-aged cohorts report more disabilities than previous cohorts (Beller & Epping, 2021). As the results of the present study suggested that grip strength is the strongest predictor of future disability, the incidence of ADL and IADL limitations may even increase when the current middle-aged cohorts get older. In addition, individuals with both weakness and cognitive impairment are at the highest risk for limitations (McGrath et al., 2020) and this interaction between different indicators should be investigated in future studies.

The exception in the present study was the sample from Greece in which none of the functional capacity predictors was associated with developing difficulties in ADL or IADL. The characteristics of the Greek sample, such as loss to follow-up, incidence of ADL or IADL limitations, or the level of functional capacity at baseline, were in line with the other countries and not likely to explain the results. One possible explanation is that during the follow-up period Greece was in the middle of an economic crisis that caused a health coverage gap in which almost one fourth of Greeks lost their health insurance coverage and access to publicly provided services (Economou et al., 2017). As economic crises are likely to increase all-cause mortality (Falagas et al., 2009), it can be speculated that in an environment with limited access to services and greater economic hardship people die before developing ADL and IADL difficulties. It would be important to replicate the analysis with future data releases to see whether this finding would change as the economic crisis attenuate.

Country-level moderator effects in the present study were mainly explained by Greece, but those that were found with and/or without Greece support the alternative hypothesis of a weaker role for functional capacity when services and resources are limited. For example, the association between word recall and IADL was weaker in countries with lower GDP and lower service capacity and access. This was also seen as a weaker association in eastern European countries (Czech Republic, Estonia, Hungary, Poland, and Slovenia) compared to other countries. This finding may indicate that in countries with better financial resources and formal services, the experience of limitations in daily activities is a more direct outcome of limited functional capacity. In the other words, the larger discrepancy between functional capacity and limitations in daily activities in countries with the less favorable environment may indicate the role of environmental challenges in the experience of disability (Guralnik & Ferrucci, 2003).

The moderator results also suggested some geographic differences that were not as clearly explained by GDP and service capacity and access. The associations between grip strength and temporal orientation and ADL limitations were stronger in northern Europe countries, namely, Denmark and Sweden, compared to other countries. Denmark and Sweden have both Nordic welfare state models with tax-funded health care systems and good service coverage (Laugesen et al., 2021). Older adults in northern countries receive more formal and less informal care compared to continental and southern Europe (Brenna & Di Novi, 2016), and also live alone more often (Mudrazija et al., 2020). A supportive environment does not only mean built but also the social environment, such as a support network (Mouchaers et al., 2022). It is possible that in northern countries, older adults receive less social support from family and informal care givers and therefore the development of difficulties is more determined by an individual’s own functional capacity. However, Nordic countries have also a high life expectancy and it is likely that in these countries people live long enough to develop limitations in daily activities. We did not account for this aspect in our analysis. Further studies among samples other than Western countries could shed more light on the role of functional capacity in less supportive environments and across countries in larger variation in life expectancy.

Since differences between countries were small, which suggested a relatively consistent role of functional capacity in developing ADL and IADL limitations, practical implications likely generalize across Western countries. A key policy priority should be preventive actions that focus on lifestyle factors throughout the life course, as they play a major role in later life functional capacity (Chatterji et al., 2015). In addition, monitoring functional capacity indicators in health care may help to identify older people who are at higher risk for disability and could benefit from intervention (Vermeulen et al., 2011). These interventions should be multi-component and individually-tailored to meet individual needs and preferences (Beswick et al., 2008; Orellano et al., 2012).

The major strength of this study is the large sample size of middle-aged and older adults from 19 countries, which permitted the generalization of findings to different Western countries. The present study appears to have the largest sample size and number of countries compared with previous studies that addressed this question. The use of harmonized sister-studies SHARE and HRS with similar methods provides the opportunity for country-level comparisons. Even though performance-based measures of functional capacity are not free of measurement error, the use of standardized protocols to assess functional capacity provides explicit indicators for country-level comparisons.

Both SHARE and HRS samples are representative of their target population aged 50 and older (Börsch-Supan et al., 2005, 2013; Sonnega et al., 2014). Specific methods, like oversampling and targeted recruitment, are used to prevent selection bias. Other advantages of these two studies are that both studies perform interviews with a close person after a participant’s death. In the present study, the inclusion of participants who moved to long-term care or died during a follow-up is an important advantage. For example, approximately half of the participants who died during the follow-up had difficulties in ADL and/or IADL before their death for the first time within the follow-up period.

Even though the whole samples of SHARE and HRS are representative of the population aged 50 and older, the sample used in the present study is not likely to be completely representative. First, 12–16% of participants were excluded from the analysis due to difficulties in ADL or IADL at baseline, and thus, the most disabled participants were excluded from the present study (as the focus was on the development of new limitations). Second, the attrition analysis suggested that, even within this healthier sample without ADL/IADL limitations at the baseline, participants with poorer functional capacity at baseline were more likely to drop out of the study. These selection and attrition biases may lead to underestimation of the impact of functional capacity on developing ADL and IADL limitations. Despite these limitations, the samples used in the present study are likely to be more diverse and representative compared to convenience samples.

This study has some other limitations as well. The samples collected in each country have different demographic statistics, and participations rates, recruitment strategies, and mortality varied between countries. There were differences also in the amount of follow-up information varying from losing only ∼2.5% of the HRS sample due to missing follow-up information on ADL or IADL to losing almost 40% of the sample in Germany and Hungary. These differences in attrition likely explain a large part of the variation in incidence rates between countries. With separate analyses for each country adjusted by demographics, the differences between samples should not substantially bias estimates.

Difficulties in ADL and IADL were assessed as a binary variable that indicated difficulty in any ADL or IADL activity. Focusing on separate activities or the number of difficulties may reveal different insights. In addition, there are micro- and meso-level environmental factors that may affect the experience of disability, such as accessibility, usability, living arrangements, and social support (Danielewicz et al., 2017; Ćwirlej-Sozańska et al., 2019). This study aimed to approach the environment from macro-level viewpoint; future work could address micro- and meso-level environmental factors, in addition to macro-level factors.

In conclusion, the results from SHARE and HRS provided strong evidence for an association between functional capacity and ADL and IADL limitations among middle-aged and older adults in Europe, Israel, and the US. The results were consistent across different functional capacity indicators, across outcomes (ADL and IADL limitations), and across countries. Good functional capacity is an important resource for maintaining independence in daily activities, regardless of country of residence.

## Supplemental Material

Supplemental Material - Functional Capacity and Difficulties in Activities of Daily Living From a Cross-National Perspective Click here for additional data file. Supplemental Material for Functional Capacity and Difficulties in Activities of Daily Living From a Cross-National Perspective by Tiia Kekäläinen, Martina Luchetti, Angelina Sutin, and Antonio Terracciano in Journal of Aging and Health.
