# Supplementary material for: Functional Capacity and Difficulties in Activities of Daily Living From a Cross-National Perspective
Source: J Aging Health. 2022 Oct 15;35(5-6):356–69. doi: 10.1177/08982643221128929 (PMC10104963; doi:10.1177/08982643221128929)
Supplement: Supplemental Material - Functional Capacity and Difficulties in Activities of Daily Living From a Cross-National Perspective [file sj-pdf-1-jah-10.1177_08982643221128929.pdf]

Functional Capacity and Difficulties in Activities of Daily Living from a Cross-National Perspective

Supplementary materials

Table S1. Comparison of the participants with and without follow-up information.

	ADL								IADL								
	Follow-up data available					Follow-up data not available			Follow-up data available					Follow-up data not available			
Country	%	N	F %	Age	Education	F %	Age	Education	%	N	F %	Age	Education	F %	Age	Education
Austria	84.3	4118	57.0	64.7±9.3	3.2±1.3[a]	58.5	64.6±9.6	3.1±1.3	83.0	3984	56.3	64.4±9.0	3.3±1.3[a]	56.6	64.9±9.6	3.1±1.2
Belgium	79.3	4131	53.6	62.9±9.7[a]	3.1±1.5[a]	50.4	61.2±9.8	2.8±1.5	78.5	4143	52.9[a]	62.7±9.5[a]	3.1±1.5[a]	48.9	62.1±9.8	2.8±1.5
Czechia	73.3	4448	56.9	64.2±8.9[a]	2.6±1.2[a]	55.1	63.0±9.5	2.5±1.1	72.0	4269	56.2[a]	63.8±8.6[a]	2.7±1.2[a]	52.9	63.0±9.5	2.6±1.1
Denmark	83.4	2336	52.1	62.1±9.9	3.5±1.4[a]	53.0	62.4±10.0	3.2±1.4	82.2	2272	51.9	61.8±9.6[a]	3.6±1.4[a]	51.7	62.9±10.3	3.3±1.4
Estonia	93.7	5196	59.2[a]	65.5±9.5[a]	3.3±1.2	53.1	62.3±10.0	3.4±1.2	91.0	5000	58.7[a]	64.9±9.2	3.4±1.2	47.7	64.8±10.8	3.3±1.2
France	77.3	4459	55.9	63.9±10.0	2.6±1.7[a]	53.1	63.7±10.7	2.4±1.7	76.5	4410	54.7	63.6±9.8	2.6±1.7[a]	51.7	63.8±10.6	2.4±1.7
Germany	60.1	1482	52.1	63.5±8.4[a]	3.5±1.1[a]	54.1	65.1±9.4	3.3±1.1	60.6	1515	51.7	63.6±8.3[a]	3.5±1.1[a]	53.7	65.0±9.3	3.4±1.1
Greece	78.9	2329	53.8	64.2±9.7	2.1±1.6	52.3	63.6±9.7	2.1±1.6	78.7	2096	50.5	63.3±9.3	2.3±1.6	50.9	62.5±9.0	2.2±1.6
Hungary	62.9	1627	57.2	63.8±8.6	3.1±1.1	53.5	64.1±9.4	3.2±1.1	63.1	1505	54.4	63.2±8.1	3.1±1.0[a]	50.3	63.1±8.7	3.2±1.1
Israel	91.4	1824	56.1	66.4±8.8[a]	3.0±1.6	57.0	64.0±9.1	3.1±1.6	87.9	1400	54.0	65.7±8.4	3.3±1.5	48.4	65.3±9.5	3.2±1.6
Italy	80.0	3164	52.5	63.9±8.7	2.0±1.2[a]	53.7	64.1±9.8	2.2±1.3	79.1	2963	51.0	63.5±8.5	2.0±1.2[a]	52.7	64.1±9.9	2.2±1.3
Netherlands	74.5	2568	54.2	63.1±9.0	3.0±1.4[a]	51.1	63.6±10.1	2.8±1.4	73.5	2471	53.5	62.9±8.9[a]	3.0±1.4[a]	50.8	63.8±10.0	2.8±1.4
Poland	75.9	1540	53.6	62.2±9.0	2.4±1.3	54.3	62.9±9.5	2.3±1.3	75.7	1575	52.3	61.6±8.6	2.5±1.3[a]	53.9	62.4±9.1	2.3±1.3
Portugal	84.3	1342	52.7	63.6±8.9	1.7±1.4[a]	56.8	64.6±9.9	2.1±1.6	85.0	1328	52.5	63.4±8.7	1.7±1.4[a]	53.6	63.9±9.9	2.2±1.6
Slovenia	87.0	2095	57.5[a]	65.1±9.8[a]	2.9±1.2[a]	49.7	62.4±9.2	3.0±1.2	85.9	2061	56.2[a]	64.5±9.5[a]	2.9±1.2	47.8	63.0±9.6	3.1±1.2
Spain	88.8	3377	52.8	65.2±10.0[a]	1.6±1.4[a]	50.7	62.7±10.2	2.0±1.4	86.6	3188	51.7	64.6±9.8	1.7±1.4[a]	47.9	64.0±10.8	2.0±1.4
Sweden	82.4	2277	53.4	66.0±9.3	2.8±1.6[a]	54.4	65.2±9.7	2.7±1.6	80.5	2240	52.9	65.4±8.9	2.8±1.6[a]	52.0	66.2±9.9	2.6±1.6
Switzerland	82.0	3109	53.3	63.9±9.7	3.2±1.1	52.9	63.8±10.4	3.1±1.2	81.7	3090	52.8	63.7±9.5	3.2±1.1	51.2	64.2±10.6	3.1±1.2
USA	97.6	13 294	58.2	67.8±9.7	2.2±1.5	56.1	68.5±10.7	2.1±1.6	97.5	13 558	58.4	67.9±9.7[a]	2.2±1.5	55.8	69.1±11.0	2.1±1.5
Total	83.3	64 716	55.7[a]	65.0±9.6[a]	2.67±1.5[a]	53.5	63.7±9.9	2.71±1.4	82.5	63 068	55.0[a]	64.7±9.7[a]	2.70±1.5[a]	51.7	63.8±9.9[a]	2.73±1.4

Note. Unadjusted mean ± standard deviations or percentages are reported. F= Female. [a]Statistically significant (p<.05) difference between "Follow-up data available" and "Follow-up data not available" groups. Differences between groups analyzed by independent samples t-test or x^2-test.

Table S2. Association between baseline functional capacity and availability of follow-up ADL information.

Country	Grip strength		Peak flow		Word recall		Temporal orientation	
	OR	95% CI	OR	95% CI	OR	95% CI	OR	95% CI
Austria	1.08	.96–1.21	1.29***	1.17–1.42	1.19***	1.10–1.29	1.42**	1.10–1.85
Belgium	1.06	.96–1.17	1.12*	1.01–1.24	1.13**	1.05–1.21	1.05	.87–1.26
Czechia	.99	.91–1.08	1.23***	1.14–1.32	1.23***	1.16–1.30	1.23*	1.04–1.46
Denmark	1.35***	1.16–1.58	1.29***	1.14–1.45	1.25**	1.13–1.39	1.24	.93–1.67
Estonia	1.28**	1.10–1.48	1.12	.99–1.25	1.16**	1.04–1.29	1.52**	1.16–1.00
France	1.10	1.00–1.22	1.11**	1.03–1.21	1.16***	1.09–1.24	1.11	.93–1.32
Germany	1.17*	1.03–1.33	1.09	.98–1.20	1.08	1.00–1.18	1.19	.89–1.60
Greece	.98	.86–1.12	1.00	.90–1.11	.95	.86–1.04	1.05	.70–1.57
Hungary	1.10	.97–1.25	1.13*	1.02–1.26	1.02	.93–1.12	1.14	.90–1.44
Israel	1.00	.79–1.26	1.14	.95–1.37	1.07	.91–1.26	1.50	.99–2.25
Italy	.93	.82–1.05	1.09	.99–1.20	.83***	.77–.91	1.20	.93–1.54
Netherlands	1.10	.98–1.24	1.19***	1.98–1.31	1.32***	1.22–1.43	1.25	1.00–1.57
Poland	1.32**	1.12–1.54	1.20**	1.05–1.36	1.01	.91–1.13	1.24	.91–1.69
Portugal	.63***	.51–.77	.92	.78–1.09	.81**	.70–.94	1.29	.84–1.97
Slovenia	.96	.79–1.15	.97	.84–1.12	1.02	.90–1.15	1.07	.79–1.45
Spain	.89	.77–1.01	.93	.83–1.03	.98	.89–1.08	.88	.68–1.14
Sweden	1.26**	1.08–1.47	1.10	.98–1.25	1.31***	1.18–1.45	1.23	.89–1.71
Switzerland	1.07	.95–1.22	1.08	.98–1.19	1.30***	1.19–1.42	1.38*	1.04–1.85
USA	1.31**	1.11–1.56	1.20*	1.04–1.38	1.23***	1.11–1.37	1.16	0.84–1.61

Note. Odds ratios (OR) and 95% confidence intervals (CI) are reported. Country-specific Z-scores of grip strength, peak flow, and word recall were used. Reference category in temporal orientation is low temporal orientation. All models are adjusted for gender. *P < .05, **P <.01, ***P <.001.

Table S3. Association between baseline functional capacity and availability of follow-up IADL information.

Country	Grip strength		Peak flow		Word recall		Temporal orientation	
	OR	95% CI	OR	95% CI	OR	95% CI	OR	95% CI
Austria	1.00	.89–1.12	1.23***	1.12–1.35	1.21***	1.12–1.31	1.29	.99–1.68
Belgium	1.06	.96–1.17	1.01	.93–1.09	1.12**	1.05–1.20	1.05	.87–1.27
Czechia	.95	.87–1.03	1.24***	1.16–1.33	1.20***	1.13–1.27	1.29**	1.10–1.52
Denmark	1.21*	1.04–1.41	1.20**	1.06–1.35	1.16**	1.05–1.28	1.11	.82–1.49
Estonia	.87	.74–1.02	.91	.80–1.03	.99	.89–1.11	1.30	.94–1.80
France	1.06	.97–1.17	1.08*	1.00–1.17	1.12***	1.06–1.20	.99	.83–1.19
Germany	1.17	1.03–1.32	1.09	.99–1.21	1.10*	1.02–1.20	1.22	.91–1.62
Greece	.96	.85–1.09	.99	.89–1.09	.95	.87–1.03	1.15	.82–1.62
Hungary	1.10	.98–1.23	1.13*	1.03–1.24	1.05	.97–1.14	1.06	.85–1.31
Israel	.79	.62–1.01	.97	.80–1.18	.95	.81–1.11	1.23	.82–1.85
Italy	.88*	.78–.99	1.05	.95–1.16	.81***	.75–.87	1.18	.93–1.50
Netherlands	1.12	1.00–1.25	1.20***	1.10–1.31	1.31***	1.21–1.41	1.25*	1.01–1.57
Poland	1.30**	1.12–1.50	1.20**	1.07–1.36	1.05	.95–1.17	1.30	.95–1.77
Portugal	.64**	.54–.78	.95	.82–1.10	.85	.74–.97	1.48	1.00–2.19
Slovenia	.86	.72–1.04	.91	.80–1.05	.96	.85–1.08	1.05	.78–1.43
Spain	.78***	.68–.90	.84**	.76–.93	.90*	.81–1.00	.82	.62–1.07
Sweden	1.08	.93–1.26	1.01	.89–1.14	1.24***	1.12–1.37	1.16	.82–1.63
Switzerland	1.01	.89–1.14	1.05	.95–1.16	1.28***	1.17–1.39	1.47**	1.11–1.96
USA	1.31**	1.11–1.56	1.97*	1.04–1.38	1.23***	1.11–1.37	1.40*	1.03–1.90

Note. Odds ratios (OR) and 95% confidence intervals (CI) are reported. Country-specific Z-scores of grip strength, peak flow, and word recall were used. Reference category in temporal orientation is low temporal orientation. All models are adjusted for gender. *P < .05, **P <.01, ***P <.001.

Table S4. Functional capacity and difficulties in ADL over 14 years follow-up.

Country	Grip strength		Peak flow		Word recall		Temporal orientation	
	HR	95% CI	HR	95% CI	HR	95% CI	HR	95% CI
Austria	.66***	.58–.75	.81***	.73–.89	.85***	.78–.93	1.17	.92–1.48
Belgium	.70***	.62–.78	.83***	.76–.91	.80***	.74–.86	1.35***	1.15–1.57
Czechia	.65***	.59–.72	.99	.92–1.06	.86***	.81–.92	1.08	.92–1.27
Denmark	.54***	.45–.64	.66***	.58–.75	.85**	.77–.94	1.63***	1.30–2.04
Estonia	.67***	.61–.74	.78***	.72–.84	.80***	.75–.85	1.17	1.00–1.38
France	.65***	.57–.73	.85***	.77–.92	.80***	.75–.87	1.12	.96–1.33
Germany	.81*	.67–.98	.88	.77–1.01	.78***	.70–.87	1.30	.91–1.85
Greece	1.01	.83–1.22	1.10	.97–1.23	1.07	.95–1.20	1.23	.91–1.67
Hungary	.66***	.55–.80	.65***	.55–.77	.71***	.62–.82	.95	.72–1.27
Israel	.73**	.61–.87	.75***	.65–.88	.82**	.73–.92	1.42**	1.11–1.82
Italy	.69***	.62–.78	.92	.84–1.01	.84***	.77–.92	1.21*	1.00–1.46
Netherlands	.64***	.52–.79	.66***	.57–.77	.77***	.70–.90	1.22	.89–1.66
Poland	.76**	.65–.89	.88*	.78–.99	.96	.86–1.07	1.18	.88–1.58
Portugal	.74**	.61–.89	.91	.80–1.02	.91	.81–1.03	1.40*	1.04–1.88
Slovenia	.70***	.58–.84	.78***	.68–.91	.83**	.74–.94	1.15	.91–1.44
Spain	.70***	.62–.78	.88**	.81–.96	.91*	.83–.98	1.31***	1.13–1.52
Sweden	.63***	.53–.74	.73***	.65–.82	.76***	.69–.83	1.37*	1.07–1.77
Switzerland	.69***	.58–.82	.76***	.67–.86	.74***	.67–.82	1.06	.78–1.46
USA	.72***	.68–.76	.75***	.72–.77	.82***	.79–.84	1.19***	1.11–1.28
Meta-analysis	.69***	.66–.71	.82***	.77–.87	.83***	.80–.86	1.20***	1.15–1.25
I^2(%)	32.08		86.69		69.73		0	
Meta-analysis without Greece	.68***	.66–.71	.80***	.76–.85	.82***	.80–.85	1.20***	1.15–1.25
I^2(%)	20.59		82.96		49.93		0	

Note. Hazard ratios (HR) and 95% confidence intervals (CI) are reported. N varies across the models due to the missing values on predictors. Z-scores of grip strength, peak flow, and word recall were used. Reference category in temporal orientation is normal temporal orientation. All models are adjusted for baseline age, gender, education, marital status, chronic diseases, and depression. Models with grip strength are also adjusted with weight and height, and models with peak flow with height. *P < .05, **P <.01, ***P <.001.

Table S5. Functional capacity and difficulties in IADL over 14 years follow-up.

Country	Grip strength		Peak flow		Word recall		Temporal orientation	
	HR	95% CI	HR	95% CI	HR	95% CI	HR	95% CI
Austria	.68***	.61–.77	.80***	.73–.88	.78***	.72–.85	1.24	.99–1.55
Belgium	.77***	.68–.87	.81***	.74–.80	.78***	.72–.84	1.39***	1.18–1.64
Czechia	.68***	.61–.76	.93	.86–1.00	.87***	.81–.93	1.31**	1.11–1.54
Denmark	.57***	.47–.69	.66***	.58–.751	.80***	.72–.88	1.48**	1.16–1.88
Estonia	.71***	.65–.78	.77***	.72–.83	.82***	.77–.87	1.09	.92–1.28
France	.57***	.50–.66	.81***	.74–.89	.79***	.73–.86	1.17	.98–1.40
Germany	.65***	.53–.80	.89	.76–1.04	.76***	.68–.86	1.34	.94–1.95
Greece	1.04	.90–1.21	1.00	.90–1.11	1.04	.94–1.15	.95	.69–1.31
Hungary	.66***	.55–.79	.69***	.59–.80	.81**	.71–.92	1.17	.89–1.54
Israel	.66***	.54–.81	.79**	.68–.92	.75***	.66–.85	1.46**	1.10–1.93
Italy	.68***	.60–.78	.87**	.78–.96	.77***	.70–.84	1.24	1.00–1.55
Netherlands	.61***	.50–.74	.75***	.65–.86	.81**	.72–.92	1.27	.98–1.70
Poland	.73***	.63–.86	.85*	.74–.97	.88*	.78–.98	1.41*	1.07–1.86
Portugal	.50***	.40–.63	.78**	.66–.93	.78***	.67–.90	1.54*	1.10–2.14
Slovenia	.64***	.54–.76	.82**	.72–.93	.81***	.72–.91	1.04	.83–1.29
Spain	.74***	.67–.83	.88**	.81–.95	.85***	.79–.92	1.25**	1.08–1.44
Sweden	.67***	.56–.80	.78***	.69–.89	.75***	.68–.83	1.12	.84–1.50
Switzerland	.65***	.54–.78	.79***	.69–.89	.77***	.69–.86	1.13	.82–1.55
USA	.70***	.66–.73	.79***	.76–.82	.77***	.74–.79	1.28***	1.20–1.37
Meta–analysis	.68	.64–.72	.81	.78–.85	.81***	.78–.83	1.24***	1.18–1.29
I^2(%)	73.09		71.50		61.43		8.02	
Meta-analysis without Greece	.67	.64–.70	.80	.77–.83	.80***	.78–.82	1.25***	1.19–1.30
I^2(%)	48.61		61.92		32.07		1.54	

Note. Hazard ratios (HR) and 95% confidence intervals (CI) are reported. N varies across the models due to the missing values on predictors. Z-scores of grip strength, peak flow, and word recall were used. Reference category in temporal orientation is normal temporal orientation. All models are adjusted for baseline age, gender, education, marital status, chronic diseases, and depression. Models with grip strength are also adjusted with weight and height, and models with peak flow with height. *P < .05, **P <.01, ***P <.001.

Table S6. The moderator effect of the drop-our rate on the associations between functional capacity indicators and ADL/IADL.

	ADL			IADL		
	Coefficient	95% CI	p	Coefficient	95% CI	p
Grip strength * Drop-out rate	.001	-.003–.003	.923	.001	-.004–.005	.814
Peak flow * Drop-out rate	-.001	-.007–.004	.602	-.001	-.004–.002	.450
Word recall * Drop-out rate	.001	-.002–.005	.492	-.001	-.005–.003	.587
Temporal orientation * Drop-out rate	.003	-.003–.008	.333	-.0001	-.007–.006	.964

Note. Coefficients are the interaction between the drop-out rate and the predictor (grip strength, peak flow, word recall, or temporal orientation) on each outcome (ADL or IADL). CI=Confidence interval.

Moderator	Grip strength				Peak flow				Word recall				Temporal orientation			
	ADL		IADL		ADL		IADL		ADL		IADL		ADL		IADL	
	Coef.	95% CI	Coef.	95% CI	Coef.	95% CI	Coef.	95% CI	Coef.	95% CI	Coef.	95% CI	Coef.	95% CI	Coef.	95% CI
GDP	-.001	-.002; .001	-.001	-.003; .002	-.002	-.005; .000	-.001	-.003; .001	-.001	-.003; .000	-.002***	-.003; -.001	.002	-.002; .006	.002	-.001; .006
Health exp.	-.005	-.002; .011	-.004	-.016; .009	-.009	-.029; .011	-.005	-.018; .008	-.004	-.014; .006	-.007**	-.011; -.003	-.010	-.017; .019	.009	-.006; .024
Service cap.	.000	-.006; .006	-.003	-.010; .005	-.008	-.018; .002	-.004	-.011; .004	-.003	-.009; .002	-.005*	-.009; -.001	.015*	.000; .030	.005	-.009; .020
Geographic area																
Northern	-.139**	-.221; -.058	-.082	-.232; .067	-.051	-.250; .148	-.068	-.202; .066	-.018	-.122; .086	.007	-.053; .067	.305*	.039; .571	.003	-.258; .264
Central	-.041	-.097; .016	-.044	-.164; .078	.053	-.119; .225	.019	-.093; .130	-.020	-.104; .065	.017	-.021-.054	.014	-.123; .150	-.027	-.177; .123
Eastern	-.046	-.102; .010	-.014	-.135; .108	.077	-.098; .253	.029	-.083; .141	.015	-.072; .101	.073***	.034; .112	-.084	-.215; .048	-.119	-.261; .024
Southern	-.015	-.078; .048	-.045	-.171; .082	.124	-.056; .305	.055	-.064; .174	.052	-.039; .144	.033	-.014; .079	.113	-.042; .266	.009	-.157; .175

Table S7. The results of country-level moderator analyses without Greece.

Note. Coef.=Coefficient, CI=Confidence interval, Health exp.=Health expenditure, Service cap.= Service capacity and access. *p<.05, **p<.01, ***p<.001

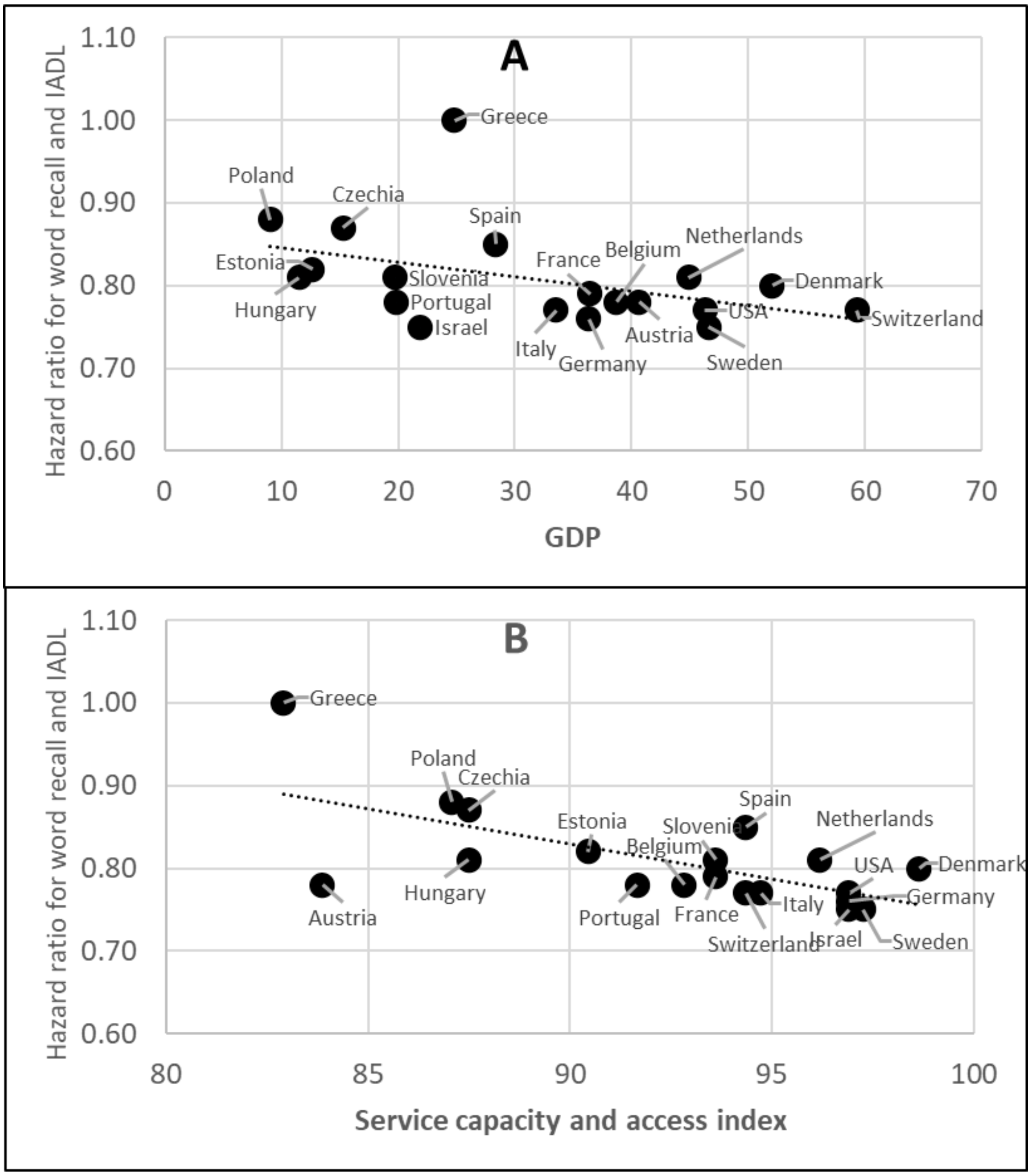

Figure S1. The moderator effect of GDP (A) and service capacity and access index (B) on the association between word recall and IADL.
